# Parental perspectives long term after neonatal clinical trial participation: a survey

**DOI:** 10.1186/s13063-020-04787-0

**Published:** 2020-11-02

**Authors:** Thomas Salaets, Emilie Lavrysen, Anne Smits, Sophie Vanhaesebrouck, Maissa Rayyan, Els Ortibus, Jaan Toelen, Laurence Claes, Karel Allegaert

**Affiliations:** 1 Department of Development and Regeneration, KULeuven, Leuven, Belgium; 2 Department of Internal Medicine and Pediatrics, UGent, Ghent, Belgium; 3 Faculty of Psychology and Educational Studies, Unit of Clinical Psychology, KULeuven, Leuven, Belgium; 4 Department of Pharmaceutical and Pharmacological Sciences, KULeuven, Leuven, Belgium; 5 Department of Hospital Pharmacy, Erasmus MC, Rotterdam, The Netherlands

**Keywords:** Neonatal clinical trials, Clinical bio-ethics

## Abstract

**Background:**

Although recruiting newborns is ethically challenging, clinical trials remain essential to improve neonatal care. There is a lack of empirical data on the parental perspectives following participation of their neonate in a clinical trial, especially at long term. The objective of this study is to assess experiences and emotions of parents, long term after trial participation in an interventional drug trial.

**Methods:**

Parents of former participants of five neonatal interventional drug trials were surveyed at long term (3–13 years ago) after participation. The survey assessed parental contentment with trial participation, perceived influence of the trial on care and health, emotional consequences of participation, and awareness of typical clinical trial characteristics on 6-point Likert scales.

**Results:**

Complete responses were received from 123 parents (52% of involved families). Twenty percent of parents did not remember participation. Those who remembered participation reported high contentment with overall trial participation (median 5.00), but not with follow-up (median 3.00). Most parents did not perceive any influence of the trial on care (median 2.00) and health (median 2.43). Almost all parents reported satisfaction and pride (median 4.40), while a minority of parents reported anxiety and stress (median 1.44) or guilt (median 1.33) related to trial participation. A relevant minority was unaware of typical trial characteristics (median 4.20; 27% being unaware).

**Conclusions:**

Overall, parents reported positive experiences and little emotional distress long term after participation. Future efforts to improve the practice of neonatal clinical trials should focus on ensuring effective communication about the concept and characteristics of a clinical trial during consent discussions and on the follow-up after the trial.

## Background

Many drugs are still being used off-label in neonates, without adequate evidence [1]. Clinical trials are necessary to identify better care practices, uncover useless or harmful therapies, and reveal new knowledge gaps [2]. Balancing the interests of future patients against the interests of an individual study participant remains a difficult ethical exercise [3]. Clinical trial participants are exposed to a treatment of which safety and efficacy are not yet established. To deliver reliable and unbiased information, participants included in a typical clinical trial are randomly and blindly assigned to either a study or control (placebo) group. This concept of a clinical trial does not necessarily serve the individual interests of a study participant.

Clinical research in neonates has specific challenges. Enrollment in trials in the neonatal intensive care unit (NICU) often occurs at a moment when parents are overwhelmed by stress and emotions. The validity of informed consent in this population has therefore been questioned [4, 5].

There is a lack of empirical data on how parents evaluate participation in a neonatal drug trial and on how the trial affects their emotional well-being [6]. Currently available studies focused on smaller non-pharmacological experimental interventions and on the parental experience during the trial procedures [7–11]. In the current study, we assessed the perspectives and emotions of parents long term after participation in an interventional drug trial conducted in the NICU. Parental understanding of the trial was assessed and neonatal outcome data collected in order to evaluate possible correlations.

## Methods

Parental perspectives were evaluated through an online questionnaire that was approved by the Ethical Committee of the University Hospitals Leuven. Formal informed consent to participate in this study was asked in the opening question of the questionnaire.

### Setting

All five interventional clinical trials, running in the NICU in University Hospitals Leuven between 2005 and 2015, in which neonates were exposed to a novel treatment (novel product or novel dosing regimen) were selected. The SMOF trial was a double-blinded, randomized controlled clinical trial comparing a new lipid emulsion to a conventional soy emulsion for parenteral nutrition in preterm infants [12]. NIRTURE was a multicenter open-label, randomized controlled trial evaluating the effect of early insulin therapy on mortality and sepsis in very low birth weight infants [13]. The DORIPENEM study was a multicenter phase I clinical trial evaluating the pharmacokinetics, safety, and tolerance of a single dose of intravenous infusion of doripenem in pre (term) neonates [14]. The LAIF-trial was a multicenter double-blinded, placebo-controlled, randomized trial to test the efficacy of adding recombinant bile salt-stimulated lipase to the feeding of preterm infants to promote growth [15]. NEOPROP was a dose-finding study to evaluate the safety and efficacy of different doses of propofol for sedation during semi-elective intubation [16]. The main characteristics of the five included trials can be retrieved from their initial publications, and are summarized in Supplementary Table 1. According to Belgian law, both parents have to sign the study informed consent form. It is furthermore standard practice in our center to discuss participation in a clinical trial with both parents.

### Recruitment

Parents of infants who participated in any of the abovementioned trials were eligible. Families were excluded if they were not familiar with Dutch language or in case of loss of parental authority. Each eligible family received an invitation for an online survey by postal mail, along with two unique access codes (one for each parent). Parents were asked to complete the survey individually. Non-responding families were invited a second time by postal mail and by phone in a third attempt. If hospital records indicated the death of the child, parents were only contacted once. The survey was accessible from October 2017 until March 2018.

### Questionnaire

The items of this questionnaire were developed after reviewing literature and interviewing different stakeholders: neonatologists, psychologists, neonatal research nurses, representatives of the Flemish preemie parent alliance (VVOC [17]), and a subgroup of parents of former study participants (*n* = 3). Face validity of the resulting questionnaire (comprehensibility and completeness) was evaluated by the same stakeholder group.

The survey was formulated in Dutch, and an English translation of the items can be found in Supplementary Tables 2 and 3. A *first part* aimed to obtain background parental information. Age-adjusted quality of life of the child (or both children if twins) who participated in the trial was assessed by the validated parent-reported PEDsQL scale (Pediatric Quality of Life Inventory) [18]. In the *second part*, parents were asked whether they remembered participation in the trial. Only respondents who answered “yes” were directed to the third and fourth parts. The *third part* assessed contentment of parents with different aspects of the trial, whether the trial influenced care for and health of their child and emotional consequences through a set of 52 items. Every item was a statement on which respondents were asked to express their level of agreement on a 6-point Likert scale ranging from one (strong disagreement) to six (strong agreement). Finally, a *fourth part* inquired awareness on five characteristics of the clinical trial in which they participated. A brief, lay term, explanation of equipoise, the possibility of adverse events, placebo or control groups, blinding, and randomization, was given. Parents indicated if they were aware of these characteristics (one item), and whether knowing this causes any distress (three items). Parents were only directed to the items relevant for the specific trial they participated in.

All items of parts three and four were organized in 17 predefined scales, measuring conceptually comparable experiences or perceptions. Cronbach’s α coefficients were calculated to assess the scale reliability [19]. For part three, α’s of all scales ranged from 0.757 to 0.957, while for part four all α’s ranged from 0.639 to 0.908, indicating overall good reliability (Supplementary Table 2 and 3).

### Bayley scales of infant development

All infants with a gestational age below 32 weeks, born in the University Hospitals Leuven, are invited for neurodevelopmental follow-up, with assessment of Bayley Scales of Infant Development (BSID). Data available for study participants was retrospectively collected. We aimed to use measurements at a prematurity-corrected age of 24 months; however, any assessment was considered if otherwise not available (median 23 months; range 7–36 months). For most infants, BSID-II was used (96%). The BSID-III motor scale scores were excluded; BSID-III cognitive and language scale scores were recalculated to BSID-II mental scale scores [20].

### Data analysis

Only complete responses are included in the analysis. All analyses were performed using SPSS (version 25, IBM). Normality was assessed with Shapiro-Wilk-tests. Non-normally distributed variables are described as medians and quartiles, while normally distributed variables are described by means and standard deviations (sd). For parents of twins who both participated in the trial, we only used the lowest value of the PedsQL and BSID-II scale scores of both twins (“worst case scenario”). Construct scale score was calculated by averaging all items of a particular scale. For visual representation, scale values were binned to integers. We interpreted construct scale medians of 1–1.5, 1.5–2.5, 2.5–3.5, 3.5–4.5, 4.5–5.5, and 5.5–6, as respectively very low, low, rather low, rather high, high, and very high levels of the measured construct. Remembrance of trial participation was compared between sexes and trials with a *X*^2^ test. Scales were compared across sexes and trials using Mann-Whitney *U* and Kruskal-Wallis tests, respectively. Spearman’s *ρ* was calculated to study correlations with ordinal or continuous variables such as BSID, PEDsQL, and parental understanding of the trial.

## Results

### Respondents

**Fig. 1:**
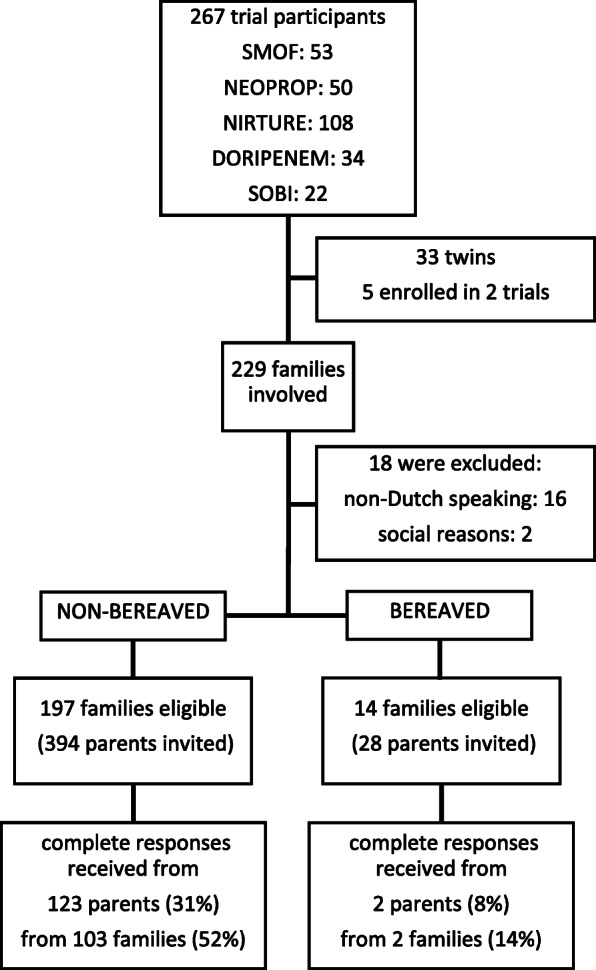
Flow chart of the study. Flow chart indicates eligibility and response rate (only considering complete responses)

A total of 194 non-bereaved and 14 bereaved families were eligible for this study. One hundred twenty-three (32%) non-bereaved (from 103 families; 53%) and two bereaved parents (from two families) completed the survey (Fig. 1). Respondents were predominantly mothers (71%), and higher education outside university was the most common educational level. The median time since participation in the trial was 11.5 (range 3.5–13.1) years. All participant characteristics are given in Table 1.

**Table 1:** Baseline characteristics of survey respondents

Baseline characteristics per respondent	
Sex, *n* (%)	
Father	36 (29)
Mother	87 (71)
Parent of twins, *n* (%)	50 (41)
Parent of twins that were both in trial, *n* (%)	24 (20)
Partner also responded to survey, *n* (%)	20 (16)
Age at completion of questionnaire, mean in years (± sd)	41.8 (± 5.6)
Age at participation in study, median in years (Q1–Q3)	31.7 (28.9–34.9)
Time since participation in study, median in years (Q1–Q3)	11.5 (5.1–12.2)
Trial, *n* (%)	
SMOF	30 (24)
NIRTURE	46 (37)
LAIF	7 (6)
DORIPENEM	9 (7)
NEOPROP	31 (25)
Education, *n* (%)	
Lower secondary education	11 (9)
Higher secondary education	31 (25)
Higher education outside university	44 (36)
Academic education (university degree)	37 (30)
BSID-II, mean (± sd)^a^	
Mental index	99.0 (± 18.5)
Motor index	95.8 (± 19.8)
Total PedsQL, median (Q1–Q3)^a^	81.7 (73.3–91.7)

Baseline characteristics of survey respondents (*n* = 123)

^a^BSID-II (Bayley scale of infant development II) or PedsQL (pediatric quality of life scale) of the respondent’s child that participated in the trial. If both members of a twin participated in the trial only the lowest value is reported

### Remembrance of trial participation

Twenty-five respondents (20%) did not remember participation of their child in a clinical trial. Remembering trial participation was not significantly correlated to sex, age, parental education, or outcome of the child (Supplementary Table 4). Surprisingly, the longer the time period since participation in the trial, the more respondents remembered participation (*ρ* = 0.202; *p* = 0.025). Remembrance was also strongly correlated to the trial (*p* = 0.016), with especially lower remembrance (61%) in NEOPROP, the most recent clinical trial (Supplementary Table 5).

### Contentment on trial participation

**Fig. 2:**
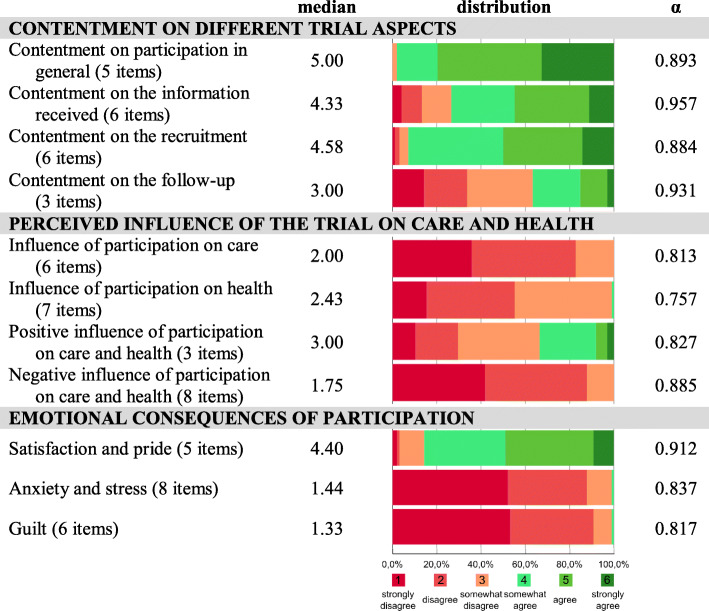
Descriptive results part I. Descriptive results of the construct scales evaluating (i) contentment on trial participation, (ii) perceived influence of the trial on care and health and (ii) emotional consequences of trial participation (n=98). Median values of construct scales are expressed on 6 point Likert scales indicating agreement from 1 (strong disagreement) to 6 (strong agreement). Color bars show relative data distribution of binned scales (%). Cronbach’s α indicates reliability of the construct scales

Overall, parents reported high levels of contentment with trial participation (median score 5.00; Fig. 2). Respondents were also rather happy with the information they received about the trial (median score 4.33); however, 26% of respondents somehow strongly disagreed. The recruitment and consent procedure of the trial was overall positively evaluated (median score 4.58). In contrast, the majority of parents (63%) was rather unsatisfied with the follow-up after the trial (median score 3.00). There were no significant correlations between contentment and sex, education, time since trial participation, trial, BSID-II, and PedsQL (Supplementary Table 6).

### Perceived influence of the trial on care and health

None of the respondents thought that trial participation affected the routine care for their child (median score 2.00; Fig. 2). Only one respondent thought that participation influenced the health of his child (median score 2.43). Thirty-four percent of respondents somewhat strongly agreed that the trial was beneficial for their child (median score 3.00), while no respondents thought it was negative (median score 1.75). Parent-reported quality of life of the child was negatively correlated to the perception that participation in the trial influenced care (*p* = 0.021), and tended to be negatively correlated to the perception that it influenced health (*p* = 0.059; Supplementary Table 6). Furthermore, the perception that participation had a positive influence on care and health was more common in participants with a lower educational attainment (*p* = 0.042) and in parents of infants with lower BSID-II mental scale scores (*p* = 0.031).

### Emotional consequences of trial participation

Rather high levels of satisfaction and pride were reported (median score 4.40; Fig. 2). Parents reported very low levels of anxiety and distress (median score 1.44) and guilt (median score 1.33). Parental education was negatively correlated to satisfaction and pride and positively correlated to guilt (respectively, *p* = 0.007 and *p* = 0.016; Supplementary Table 6).

### Awareness of typical trial characteristics

**Fig. 3:**
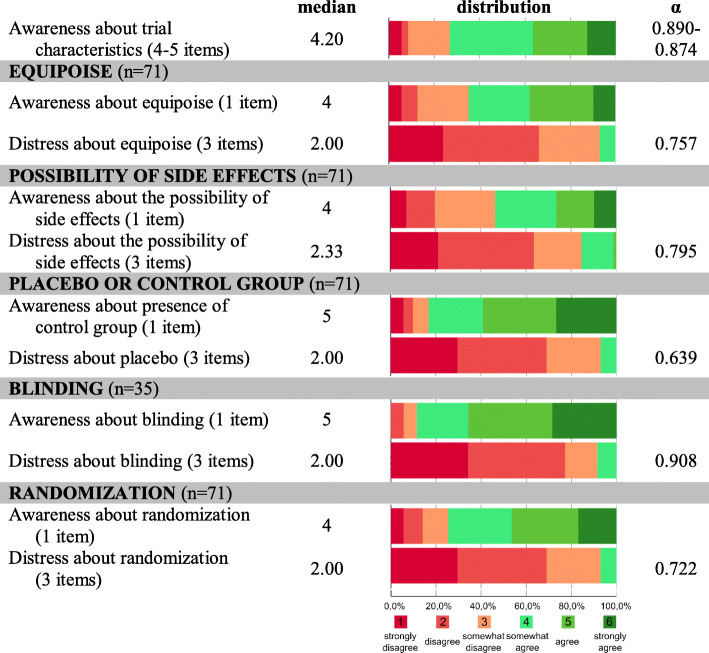
Descriptive results part II . Descriptive results of the construct scales and items evaluating awareness of and distress about typical clinical trial characteristics (n=35-71). Median values of construct scales are expressed on 6 point Likert scales indicating agreement from 1 (strong disagreement) to 6 (strong agreement). Color bars show relative data distribution of binned scales (%). Cronbach’s α indicates reliability of the construct scales

Most, but not all, parents were aware of the characteristics of the clinical trial in which their child participated (median score 4.20; 27% being unaware; Fig. 3). Looking at individual characteristics, awareness was lowest for the possibility of adverse events, followed by equipoise, randomization, the presence of a control group, and blinding. For all five trial characteristics, a large majority of respondents did not feel distressed after reading a brief explanation on the topic (medians ranging from 2 to 2.33). There was a strong negative correlation between awareness and distress for each individual characteristic (*p* ≤ 0.001; Supplementary Table 7).

Fathers were overall more aware (*p* = 0.008–0.178) and less distressed (*p* = 0.008–0.128) than mothers about the characteristics of the trial (Supplementary Table 8). Overall, awareness about typical trial characteristics tended to be related to the trial in which the respondent’s child participated (*p* = 0.0665). Being aware about the characteristics of the trial was also positively correlated with positive perspectives on trial participation, and negatively to anxiety and guilt (Supplementary Table 9).

### Bereaved parents

Only two bereaved parents responded, resulting in insufficient data for this group.

## Discussion

Overall, parents who remembered the trial looked back at the participation of their child as a positive experience. They were overall contented with their participation and reported rather high levels of satisfaction and pride and low levels of anxiety, stress, and guilt. These findings could provide comfort to researchers, who often doubt whether it is acceptable to burden the already distressed family of a sick newborn with recruitment for a clinical trial [21]. Furthermore, it is reassuring that parents of babies with worse outcome are equally comfortable with trial participation. Other studies have previously documented comparable positive experiences among parents of neonatal clinical trial perspectives [7–9, 11]. The current study however is the first to evaluate the perspectives of parents of neonates participating in interventional pharmacological trials, with a relatively invasive experimental intervention, and once the long-term outcome is known.

Interestingly, 20% of the responding parents did not remember trial participation. We observed significant differences between parents participating in the five included trials. More parents forgot participation in NIRTURE (22%) and NEOPROP (39%) in comparison to the other 3 trials. Both NIRTURE and NEOPROP required recruitment very early after birth, in often instable preterm babies. Additionally, NEOPROP involved only a very brief investigational drug exposure, and follow-up was limited at 12 h. We hypothesize that emotional distress at the time of consent and during the study procedure negatively affects remembrance of trial participation.

It has often been questioned whether emotionally distressed parents of sick newborns are able to give voluntary, competent, and informed consent for trial participation [4, 21–24]. Overall, good levels of awareness on the characteristics of the clinical trial in which their child participated indicate that most parents in this study (regardless of education) understood to what they consented. These results are comparable to earlier studies with parents of pediatric study participants, [25] but contrast qualitative work demonstrating poor understanding of the concept of a clinical trial and randomization in a sample of parents of neonatal study participants [26]. In our sample, fathers were significantly more aware and less distressed about typical trial characteristics. We hypothesize that mothers are more emotionally distressed at the moment of consent and often still bear the consequences from medical conditions leading to preterm birth that also affect cognitive functioning and possibly memory [27, 28].

Nevertheless, still, 35% and 46% of the respondents were unaware of the uncertainty concerning the effectivity (equipoise) and safety (possible adverse events) of the experimental intervention. Furthermore, 34% of respondents perceived positive effects of the clinical trial, while no respondents perceived negative effects. The disproportional optimism regarding the beneficial effects of trial participation due to a failure in discriminating the goals of a trial from normal clinical care is called therapeutic misconception [29, 30]. Our data suggests that parents with a lower educational attainment are more likely to overestimate the beneficial effects of the trial and underestimate the side effects.

Self-reported awareness of study characteristics was in our study strongly correlated to positive perspectives on trial participation. Researchers asking for consent should ensure that parents capture and retain the concept of the trial properly. Recently, professional stakeholders and parent representatives made recommendations to ensure effective communication in consent procedures in this setting [31]. Alternatively, other types of consent procedures avoiding recruitment during a stressful period (such as antenatal consent or deferred consent) could be explored [4, 32].

This study has some limitations. First, one has to be careful in generalizing our findings. It is possible that our study is biased by selection of parents at least remembering trial participation, or having a strong opinion on it. Our results on remembrance of the trial and awareness of different trial characteristics indicate that the perspectives of parents might also be different depending on incidental aspects of the specific trial. We also noted important differences between trials on remembrance of participation. Extrapolation to trials with other specific characteristics might be difficult. Second, it is possible that some results are biased by social desirability, leading to a favorable trial evaluation. A qualitative study would be able to explore how important this aspect was, and whether parents had truly in-depth understanding of the concept and risks of the trial in which they participated [26, 33]. Third, despite our focus on long-term perspectives, the long recall time (median 11.5 years) might have made it difficult to give a thought out opinion on some of the questions in the survey. Finally, with an adapted and less aggressive recruitment strategy, we were only able to recruit two bereaved parents. For the perspectives of this group, we can refer to the BRACELET study that has shown that most bereaved parents consider the trial as irrelevant to their child’s death [34].

## Conclusions

In conclusion, parents report positive perspectives and low levels of emotional distress at long term after trial participation of their neonate. This finding can reassure clinicians, researchers, and review boards who worry about the emotional effect of trial participation on parents of neonates. Future efforts to improve the practices of neonatal clinical trials should focus on ensuring effective communication of the concept, risks, and benefits of the trial to already distressed parents. Special attention should go to mothers early after birth (that were often less aware of study characteristics) and parents with lower educational attainment (at risk of therapeutic misconception).

## Supplementary information

**Additional file 1:** Supplementary Tables 1-9.

## Data Availability

The anonymized dataset used and/or analyzed during the current study is available from the corresponding author on reasonable request.

## Abbreviations

NICU: Neonatal intensive care unit
VVOC: Vlaamse Vereniging van Ouders van Couveusekinderen (Flemish preemie parent alliance)
PEDsQL: Pediatric Quality of Life Inventory
BSID: Bayley Scale of Infant Development
