# Supplementary material for: Parental perspectives long term after neonatal clinical trial participation: a survey
Source: Trials. 2020 Nov 2;21:907. doi: 10.1186/s13063-020-04787-0 (PMC7607657; doi:10.1186/s13063-020-04787-0)
Supplement: Supplementary file 1 — Additional file 1: Supplementary Tables 1-9. [file 13063_2020_4787_MOESM1_ESM.docx]

**Supplementary Table 1.** **Summary of the characteristics of the 5 interventional drug trials in neonates**. ^a^GA: gestational age; ^b^INSURE: intubation-surfactant administration-extubation; ^c^NIRS: Near Infra-Red Spectroscopy; ^d^aEEG: amplitude-integrated electro-encephalography.

|  | **SMOF** | **NIRTURE** | **DORIPENEM** | **LAIF** | **NEOPROP** |
| --- | --- | --- | --- | --- | --- |
| **Full name** (if acronym) | // | Neonatal Insuline Replacement Therapy in Europe | // | Lipase Added to Infant Feeding | Exploratory Propofol Dose Finding Study In Neonates |
| **Epoch** | 2004-2006 | 2005-2007 | 2009-2011 | 2011-2014 | 2012-2014 |
| **Patient population** | Infants <34 weeks GA^a^, 500-2000g, with an estimated requirement for parenteral nutrition of >7days | Very low birth weight infants (<1500g) | Preterm (and term) neonates <12 weeks postnatal age, at risk of infection | NICU patients, born before 32 weeks GA^a^ | NICU patients needing sedation for semi-elective intubation (mostly INSURE^b^ procedures) |
| **Intervention** | SMOFlipid 20% in parenteral nutrition | Continuous IV infusion of insulin (0.05 u/kg/h) | Single dose of IV doripenem, through separate line | rhBSSL (recombinant bile salt-stimulated lipase) added to normal feeding | Single dose of IV propofol bolus (dose finding approach) |
| **Comparison** (control group) | Conventional soy bean emulsion | Normal neonatal care | N/A | Normal feeding | N/A |
| **Blinding** | √ | open label | open label | √ | open label |
| **Randomization** | √ | √ | N/A | √ | N/A (randomized in strata) |
| **Postnatal age at start of study** | Mean = 7 days | Median = 13.4 hours (before 24h of age) | Mean = 3-52 days (in different groups) | Mean = 3.2 weeks (100ml/kg of feeding) | Mean = 33.9 hours |
| **Consent procedure** | Postnatal written informed consent | Postnatal written informed consent + continuing consent | Postnatal written informed consent | Postnatal written informed consent | Postnatal written informed consent |
| **Duration of intervention** | 7-14 days | First 7 days | 1 hour infusion | 4 weeks | 12 hours |
| **Additional procedures related to the study** | 5 additional blood samples | Subcutaneous glycaemia sensor, frequent finger pricks, 4 additional blood samples | 4 additional blood samples within 7h | 1 additional blood sample (follow-up consultation) | NIRS^c^ and aEEG^d^ monitoring during procedure  2 blood samples for PK |
| **Follow-up** | No a priori defined follow-up | No a priori defined follow-up | 7 days after infusion | 3 months after treatment and 12 months corrected age | No a priori defined follow-up |

**Supplementary Table 2.** **Descriptive results of the construct scales and individual items evaluating contentment on trial participation, perceived influence of the trial on care and health and emotional consequences of trial participation** (n=98). Median and quartiles values are expressed as 6 point Likert scales indicating agreement from 1 (strong disagreement) to 6 (strong agreement) for all individual items, and averaged for all items in a construct. Cronbach’s α indicates reliability of the construct scales.

|  | median | Q1-Q3 | α |
| --- | --- | --- | --- |
| **CONTENTMENT ON DIFFERENT TRIAL ASPECTS** | | | |
| **Contentment on participation in general** | **5.00** | **4.60-5.80** | **0.893** |
| I am contented that I let my son/daughter participate in the trial | 5 | 5-6 |  |
| I would, if I was in the same situation, participate again in the trial. | 5 | 5-6 |  |
| I would recommend participation in the trial to other parents in a comparable situation. | 5 | 5-6 |  |
| Participation of my son/daughter in the trial was a positive experience for me. | 5 | 4-6 |  |
| Participation of my son/daughter in the trial was a negative experience for me. *(inverted)* | 2 | 1-2 |  |
| **Contentment on the information received** | **4.33** | **3.50-5.00** | **0.957** |
| I’m contented with the information I received about the trial. | 5 | 3-5 |  |
| I was, on beforehand, well informed about the course of the trial. | 4.5 | 4-5 |  |
| I was, on beforehand, well informed about the possible consequences of the trial. | 4 | 3-5 |  |
| The information forms about the trial were useful. | 4 | 3-5 |  |
| I was sufficiently informed to make a good decision about the participation of my son/daughter in the trial. | 4 | 4-5 |  |
| I could turn to someone with my questions on the trial. | 4 | 3-5 |  |
| **Contentment on the recruitment** | **4.58** | **4.17-5.17** | **0.884** |
| I’m contented with the conversation in which I was asked for participation of my son/daughter in the trial. | 5 | 4-5 |  |
| I’m contented with the way how I was asked for participation of my son/daughter in the trial. | 5 | 4-5 |  |
| I’m contented with the timing of the conversation in which I was asked for participation of my son/daughter in the trial. | 4 | 4-5 |  |
| I have the feeling I was under pressure to let my son/daughter participate in the trial. *(inverted)* | 2 | 1-2 |  |
| I think I was, at the moment when I was asked for participation of my son/daughter in the trial, capable of taking this decision. | 5 | 3.75-5 |  |
| I think the person that asked for my consent on the participation of my son/daughter in the trial was well placed to do so. | 5 | 4-5.25 |  |
| **Contentment on the follow-up** | **3.00** | **2.00-4.00** | **0.931** |
| I’m contented with the follow-up after the trial. | 3.5 | 2-4 |  |
| I think there were enough follow-up consultations after the trial. | 3 | 2-4 |  |
| I was well informed about the results of the trial. | 3 | 2-4 |  |
| **PERCEIVED INFLUENCE OF THE TRIAL ON CARE AND HEALTH** | | | |
| **Influence of participation on care** | **2.00** | **1.17-2.33** | **0.813** |
| I have the feeling that participation in the trial influenced the normal care for my son/daughter. | 2 | 1-2 |  |
| I think the quality of the care for my son/daughter decreased because of participation in the trial.† | 1 | 1-2 |  |
| I think my son/daughter received better care because of his/her participation in the trial.* | 2 | 1-3.25 |  |
| I have the feeling that infomation about the health status of my child was too much mixed with information about the trial.† | 2 | 1-2 |  |
| I have the feeling that the interest of my child was conflicted by the interest of the trial by the doctors.† | 2 | 1-2 |  |
| I have the feeling that doctors were more interested in the trial than in my child.† | 1 | 1-2 |  |
| **Influence of participation on health** | **2.43** | **1.86-2.86** | **0.757** |
| I think participation in the trial has influenced the health of my son/daughter. | 2 | 1-3 |  |
| I think my child has experienced medical benefits because of his/her participation in the trial.* | 3 | 2-4 |  |
| I think participation in the trial was good for the health of my child.* | 4 | 3-4 |  |
| I think participation in the trial was bad for the health of my trial.† | 2 | 1-3 |  |
| I think the study procedure was burdensome for my child.† | 2 | 1-3 |  |
| I think my child has experienced adverse events in the trial.† | 2 | 1-2 |  |
| I attribute the current health problems of my child to participation in the trial.† | 1 | 1-2 |  |
| **Positive influence of participation on care and health*** | **3.00** | **2.33-4.00** | **0.827** |
| **Negative influence of participation on care and health†** | **1.75** | **1.12-2.12** | **0.885** |
| **EMOTIONAL CONSEQUENCES OF PARTICIPATION** | | | |
| **Satisfaction and pride** | **4.40** | **3.80-5.00** | **0.912** |
| The participation of my son/daughter in the trial satisfies me. | 4 | 3.75-5 |  |
| I am proud that I let my son/daughter participate in the trial. | 4 | 3.75-5 |  |
| It satisfies me that the participation of my son/daughter helped advancing science. | 5 | 4-6 |  |
| It satisfies me that I, by letting my son/daughter participate in the trial, have exploited all possible medical means for my son/daughter. | 5 | 4-6 |  |
| It satisfies me that I, by letting my son/daughter participate in the trial, was able to have an important contribution myself to the health of my son/daughter. | 4 | 3-5 |  |
| **Anxiety and stress** | **1.44** | **1.12-2.00** | **0.837** |
| Participation of my son/daughter in the trial, still causes fear. | 1 | 1-2 |  |
| Participation of my son/daughter in the trial, still makes me uncertain. | 1 | 1-2 |  |
| Participation of my son/daughter in the trial, still makes me worried. | 1 | 1-2 |  |
| Participation of my son/daughter in the trial, still causes stress. | 1 | 1-2 |  |
| Participation of my son/daughter in the trial, still causes an unsafe feeling. | 1 | 1-2 |  |
| I brood sometimes on the possible consequences of participation in the trial. | 1 | 1-2 |  |
| Participation of my son/daughter in the trial does not influence my peace of mind. *(inverted)* | 5 | 3.75-6 |  |
| Trials, like the one my child participated in, are being performed to solve uncertainties in medical care. It worries me that this means that doctors are actually uncertain about many therapies. | 2 | 1-3 |  |
| **Guilt** | **1.33** | **1.17-2.00** | **0.817** |
| Participation of my son/daughter in the trial, causes a guilty feeling. | 1 | 1-2 |  |
| I feel guilty about my decision to let my son/daughter participate in the trial. | 1 | 1-2 |  |
| I regret my decision to let my son/daughter participate in the trial. | 1 | 1-2 |  |
| I have the feeling I harmed my son/daughter with my decision to let him/her participate in the trial. | 1 | 1-2 |  |
| I have the feeling I have to make up something with my son/daughter because of my decision to let him/her participate in the trial. | 1 | 1-2 |  |
| I have the feeling I did something good for my son/daughter with my decision to let him/her participate in the trial. *(inverted)* | 4.5 | 4-5 |  |

**Supplementary Table 3. Descriptive results of the construct scales and items evaluating awareness of and distress about typical clinical trial characteristics** (n=35-71). Median and quartiles values are expressed on 6 point Likert scales for all individual items, and averaged for all items in a construct. Cronbach’s α indicates reliability of the construct scales. ^a^Cronbach’s α for awareness on 4 characteristics of SMOF, NIRTURE and LAIF (without blinding) - for awareness on 5 characteristics of SMOF and LAIF.

|  | median | Q1-Q3 | α |
| --- | --- | --- | --- |
| **Feeling of being informed about typical trial characteristics*** | **4.20** | **3.50-5.00** | **0.890-0.874**^a^ |
| **EQUIPOISE** (n=71)  Clinical trials, like the one in which your child participated, are being performed to test new treatment strategies. Doctors have good reasons to investigate these treatments, but do not know for sure on beforehand if the new treatment is better than standard care. This uncertainty is the reason to perform the study. There is a possibility, that after the trial, the new treatment is less effective than standard of care. The experimental treatment that was studied in the trial in which your son/daughter participated, could thus be either better, equal or worse than the treatment that other children receive in the same situation. | | | |
| I think I was, before participation in the trial, well informed about this.* | 4 | 3-5 |  |
| **Distress about equipoise** | **2.00** | **1.67-2.67** | **0.757** |
| I am, after reading this explanation, still happy that my son/daughter participated in the trial. *(inverted)* | 5 | 4-5 |  |
| I am, after reading this explanation, worried about the participation of my son/daughter in the trial. | 2 | 1-3 |  |
| I would, after reading this explanation, fill in the first part of this questionnaire differently. | 2 | 1-3 |  |
| **POSSIBILITY OF SIDE EFFECTS** (n=71)  In a clinical trial, like the one in which your child participated, not only the effectivity, but also the safety of a new treatment is studied. No matter how small, there is always a risk that the new treatment will cause adverse reactions. Also you son/daughter ran, by participation in the trial, a theoretical risk for adverse reactions. | | | |
| I think I was, before participation in the trial, well informed about this. | 4 | 3-5 |  |
| **Distress about the possibility of side effects** | **2.33** | **2.00-3.00** | **0.795** |
| I am, after reading this explanation, still happy that my son/daughter participated in the trial. *(inverted)* | 4 | 4-5 |  |
| I am, after reading this explanation, worried about the participation of my son/daughter in the trial. | 2 | 1-3 |  |
| I would, after reading this explanation, fill in the first part of this questionnaire differently. | 2 | 1-3 |  |
| **PLACEBO/CONTROL GROUP** (n=71)  To be able to conclude that the new studied treatment is effective and safe, it is compared to other children in the same trial that receive a fake treatment (‘placebo’). Some children that participate in a trial thus receive a new experimental treatment, while others receive a fake treatment (on top of the current standard treatment of course). The fact that your son/daughter participated in the trial, thus does not mean that he/she received the new experimental treatment. | | | |
| I think I was, before participation in the trial, well informed about this. | 5 | 4-6 |  |
| **Distress about placebo** | **2.00** | **1.33-2.67** | **0.639** |
| I am, after reading this explanation, still happy that my son/daughter participated in the trial. *(inverted)* | 5 | 4-5 |  |
| I am, after reading this explanation, worried about the participation of my son/daughter in the trial. | 2 | 1-3 |  |
| I would, after reading this explanation, fill in the first part of this questionnaire differently. | 2 | 1-3 |  |
| **BLINDING** (n=35)  Sometimes it is very subjective to assess whether a drug or treatment was effective or not. To avoid that parents and doctors, based on expectations, will over- or underestimate this effect, most clinical trials are organized in such a way that both parents and doctors do not know whether a patient receives the new study treatment or a placebo treatment. This is called double blinding. This means that both you and the doctors taking care of you son/daughter did not know whether he/she received the study treatment or a fake control treatment. | | | |
| I think I was, before participation in the trial, well informed about this. | 5 | 4-6 |  |
| **Distress about blinding** | **2.00** | **1.00-2.33** | **0.908** |
| I am, after reading this explanation, still happy that my son/daughter participated in the trial. *(inverted)* | 5 | 4-6 |  |
| I am, after reading this explanation, worried about the participation of my son/daughter in the trial. | 2 | 1-3 |  |
| I would, after reading this explanation, fill in the first part of this questionnaire differently. | 2 | 1-3 |  |
| **RANDOMIZATION** (n=71)  In a clinical trial, we try to avoid that the effect of a study drug or treatment is over- or underestimated because of unequal distribution of patients. One can imagine that when all the sickest patients for instance are assigned to the control group, the effect of the study treatment (in comparison to the placebo or control treatment) can be severely overestimated. The distribution or assignment of patients to the study or control groups is therefore performed as random as possible. In clinical trials it is a computer that decides to which group a patient is assigned, based on randomness and not based on patient characteristics (health status, age, sex). This was also the case for the trial in which your child participated. | | | |
| I think I was, before participation in the trial, well informed about this. | 4 | 3-5 |  |
| **Distress about randomisation** | **2.00** | **1.33-2.67** | **0.722** |
| I am, after reading this explanation, still happy that my son/daughter participated in the trial. *(inverted)* | 5 | 4-5 |  |
| I am, after reading this explanation, worried about the participation of my son/daughter in the trial. | 2 | 1-3 |  |
| I would, after reading this explanation, fill in the first part of this questionnaire differently. | 2 | 1-3 |  |

**Supplementary Table 4. Correlations with remembrance of trial participation** (n=94-123). ^a^X²-test expressed as X²-statistic (p-value). ^b^Spearman’s ρ expressed as ρ (p-value). Light grey boxes indicate significance.

|  | **Remembrance of participation** |
| --- | --- |
| **Sex**^a^ | 0.024 (0.876) |
| **Age at completion of questionnaire in years**^b^ | -0.026 (0.776) |
| **Age at participation in study in years**^b^ | -0.134 (0.139) |
| **Education**^b^ | 0.055 (0.547) |
| **Time since participation in study in years**^b^ | 0.202 (0.025) |
| **Trial**^a^ | 12.240 (0.016) |
| **BSID-II-mental scale**^b^ (n=95) | -0.028 (0.786) |
| **BSID-II-motor scale**^b^ (n=94) | -0.102 (0.328) |
| **Total PedsQL**^b^ | -0.096 (0.292) |

**Supplementary Table 5. 5x2 table of remembrance of trial participation versus trial** (n=123). X²-test indicates significant correlation between remembrance and trial (p=0.016).

|  | **SMOF** | **NIRTURE** | **LAIF** | **DORIPENEM** | **NEOPROP** | **Total** |
| --- | --- | --- | --- | --- | --- | --- |
| **Does not remember** | 2 (7%) | 10 (22%) | 0 (0%) | 1 (11%) | 12 (39%) | 25 |
| **Remembers** | 28 (93%) | 36 (78%) | 7 (100%) | 8 (89%) | 19 (61%) | 98 |
| **Total** | 30 | 46 | 7 | 9 | 31 | 123 |

**Supplementary Table 6. Correlations with construct scales on contentment on diferent aspects of the trial, perceived influence on care and health and emotional consequences of trial participation** (n=74-98). ^a^Mann-Whitney U-test comparing male to female respondents expressed as p-value. ^b^Spearman’s ρ expressed as ρ (p-value). ^c^Kruskal-Wallis test, expressed as p-value. Light grey boxes indicate significance.

|  | **Sex**^a^ | **Educa-tion**^b^ | **Time since trial**^b^ | **Trial**^c^ | **BSID-II-mental**^b^ (n=75) | **BSID-II-motor**^b^ (n=74) | **Peds-QOL**^b^ |
| --- | --- | --- | --- | --- | --- | --- | --- |
| **CONTENTMENT ON DIFFERENT TRIAL ASPECTS** | | | | | | | |
| Contentment on participation in general | 0.456 | -0.108 (0.292) | 0.093 (0.364) | 0.104 | 0.116 (0.324) | -0.012 (0.919) | 0.146 (0.153) |
| Contentment on the information received | 0.556 | 0.009 (0.931) | -0.104 (0.306) | 0.148 | 0.037 (0.750) | 0.031 (0.795) | 0.069 (0.498) |
| Contentment on the recruitment | 0.098 | -0.091 (0.371) | -0.004 (0.966) | 0.474 | -0.007 (0.953) | -0.006 (0.959) | 0.055 (0.587) |
| Contentment on the follow-up | 0.848 | -0.109 (0.286) | -0.026 (0.799) | 0.108 | 0.009 (0.939) | 0.005 (0.963) | 0.105 (0.304) |
| **PERCEIVED INFLUENCE OF THE TRIAL ON CARE AND HEALTH** | | | | | | | |
| Influence of participation on care | 0.293 | -0.025 (0.810) | -0.076 (0.458) | 0.502 | -0.092 (0.435) | 0.016 (0.891) | -0,234 (0.021) |
| Influence of participation on health | 0.617 | -0.105 (0.305) | 0.149 (0.142) | 0.185 | -0.215 (0.065) | 0.044 (0.710) | -0,192 (0.059) |
| Positive influence of participation on care and health | 0.653 | -0.206 (0.042) | 0.056 (0.548) | 0.141 | -0.249 (0.031) | -0.002 (0.988) | -0,158 (0.119) |
| Negative influence of participation on care and health | 0.684 | 0.030 (0.770) | -0.025 (0.810) | 0.515 | -0.045 (0.702) | 0.017 (0.887) | -0,165 (0,105) |
| **EMOTIONAL CONSEQUENCES OF PARTICIPATION** | | | | | | | |
| Satisfaction and pride | 0.959 | -0.269 (0.007) | 0.044 (0.665) | 0.660 | 0.010 (0.931) | 0.044 (0.711) | 0,175 (0.084) |
| Anxiety and stress | 0.493 | 0.109 (0.288) | -0.087 (0.396) | 0.439 | -0.006 (0.959) | 0.151 (0.200) | -0,120 (0.238) |
| Guilt | 0.299 | 0.243 (0.016) | 0.139 (0.171) | 0.160 | 0.022 (0.852) | 0.007 (0.956) | -0,036 (0.724) |

**Supplementary Table 7. Correlations of awareness on individual clinical trial characteristics to distress about the same characteristic** (n=35-71). Spearman’s ρ expressed as ρ (p-value). Light grey boxes indicate significance.

|  | **Correlation of awareness x distress** |
| --- | --- |
| **Equipoise** | -0.579 (<0.001) |
| **Possibility of side effects** | -0.722 (<0.001) |
| **Presence of a control group** | -0.390 (0.001) |
| **Blinding** | -0.559 (<0.001) |
| **Randomization** | -0.512 (<0.001) |

**Supplementary Table 8. Correlations with construct scales and items about awareness on typical clinical trial characteristics** (n=27-71). ^a^Mann-Whitney U-test comparing male to female respondents expressed as p-value. ^b^Spearman’s ρ expressed as ρ (p-value). ^c^Kruskal-Wallis test, expressed as p-value. Light grey boxes indicate significance.

|  | **Sex**^a^ | **Educa-tion**^b^ | **Time since trial**^b^ | **Trial**^c^ | **BSID-II-mental**^b^ (n=61) | **BSID-II-motor**^b^ (n=62) | **Peds-QOL**^b^ |
| --- | --- | --- | --- | --- | --- | --- | --- |
| Awareness about typical trial characteristics | 0.018 | 0.111 (0.359) | -0.001 (0.994) | 0.065 | 0.041 (0.751) | 0.068 (0.598) | -0.030 (0.801) |
| **EQUIPOISE** (n=71) | | | | | | | |
| Awareness about equipoise | 0.178 | 0.057 (0.636) | 0.047 (0.697) | 0.327 | -0.012 (0.929) | 0.013 (0.919) | 0.112 (0.354) |
| Distress about equipoise | 0.031 | -0.007 (0.954) | 0.042 (0.728) | 0.923 | 0.034 (0.795) | 0.108 (0.403) | -0.156 (0.193) |
| **POSSIBILITY OF SIDE EFFECTS** (n=71) | | | | | | | |
| Awareness about the possibility of side effects | 0.066 | 0.057 (0.638) | 0.002 (0.985) | 0.414 | -0.146 (0.263) | -0.008 (0.954) | -0.034 (0.778) |
| Distress about the possibility of side effects | 0.016 | 0.001 (0.991) | -0.123 (0.309) | 0.817 | 0.032 (0.806) | 0.031 (0.811) | -0.129 (0.285) |
| **PLACEBO OR CONTROL GROUP** (n=71) | | | | | | | |
| Awareness about the presence of a control group | 0.018 | -0.010 (0.932) | 0.077 (0.524) | 0.042 | 0.018 (0.892) | 0.224 (0.081) | -0.043 (0.721) |
| Distress about placebo | 0.010 | -0.046 (0.703) | 0.028 (0.814) | 0.670 | 0.015 (0.907) | 0.071 (0.585) | -0.171 (0.155) |
| **BLINDING** (n=35) | | | | | | | |
| Awareness about blinding | 0.009 | 0.125 (0.474) | -0.042 (0.810) | 0.698 | 0.014 (0.946) | -0.086 (0.663) | 0.053 (0.761) |
| Distress about blinding | 0.128 | -0.224 (0.195) | 0.028 (0.875) | 0.658 | -0.095 (0.637) | 0.026 (0.896) | -0.340 (0.046) |
| **RANDOMISATION** (n=71) | | | | | | | |
| Awareness about randomization | 0.008 | 0.104 (0.389) | 0.011 (0.930) | 0.375 | -0.019 (0.884) | 0.101 (0.436) | -0.049 (0.685) |
| Distress about randomisation | 0.008 | -0.016 (0.895) | 0.065 (0.591) | 0.678 | 0.201 (0.121) | 0.063 (0.626) | -0.084 (0.486) |

**Supplementary Table 9. Correlations of awareness about typical trial characteristics to construct scales on contentment on trial participation, perceived influence on care and health and emotional consequences of participation** (n=71). Spearman’s ρ expressed as ρ (p-value). Light grey boxes indicate significance.

|  | **Awareness about typical trial characteristics** |
| --- | --- |
| **CONTENTMENT ON DIFFERENT TRIAL ASPECTS** | |
| Contentment on participation in general | 0.360 (0.002) |
| Contentment on the information received | 0.746 (<0.001) |
| Contentment on the recruitment | 0.726 (<0.001) |
| Contentment on the follow-up | 0.596 (<0.001) |
| **PERCEIVED INFLUENCE OF THE TRIAL ON CARE AND HEALTH** | |
| Influence of participation on care | 0.019 (0.876) |
| Influence of participation on health | 0.249 (0.036) |
| Positive influence of participation on care and health | 0.402 (0.001) |
| Negative influence of participation on care and health | -0.120 (0.317) |
| **EMOTIONAL CONSEQUENCES OF PARTICIPATION** | |
| Satisfaction and pride | 0.300 (0.011) |
| Anxiety and stress | -0.289 (0.015) |
| Guilt | -0.373 (0.001) |
